# An Overview of the Malaria Control Programme in Zambia

**DOI:** 10.5402/2013/495037

**Published:** 2012-12-09

**Authors:** Emmanuel Chanda, Mulakwa Kamuliwo, Richard W. Steketee, Michael B. Macdonald, Olusegun Babaniyi, Victor M. Mukonka

**Affiliations:** ^1^Directorate of Public Health and Research, National Malaria Control Centre, Ministry of Health, P.O. Box 32509, Lusaka, Zambia; ^2^Malaria Control and Evaluation Partnership in Africa, PATH, P.O. Box 900922, Seattle, WA 98109, USA; ^3^Global Malaria Programme, WHO Headquarters, World Health Organization, Avenue Appia 20, 1211 Geneva, Switzerland; ^4^WHO Country Office, World Health Organization, P.O. Box 51449, Ridgeway, Lusaka, Zambia; ^5^Department of Public Health, School of Medicine, Copperbelt University, P.O. Box 71191, Ndola, Zambia

## Abstract

The Zambian national malaria control programme has made great progress in the fight against Malaria. The country has solid, consistent, and coordinated policies, strategies, and guidelines for malaria control, with government prioritizing malaria in both the National Health Strategic Plan and the National Development Plan. This has translated into high coverage of proven and effective key preventive, curative, and supportive interventions with concomitant marked reduction in both malaria cases and deaths. The achievements attained can be attributed to increased advocacy, communication and behaviour changes, efficient partnership coordination including strong community engagement, increased financial resources, and evidence-based deployment of key technical interventions in accordance with the national malaria control programme policy and strategic direction. The three-ones strategy has been key for increased and successful public-private sector partner coordination, strengthening, and mobilization. However, maintaining the momentum and the gains is critical as the programme strives to achieve universal coverage of evidence-based and proven interventions. The malaria control programme's focus is to maintain the accomplishments, by mobilizing more resources and partners, increasing the government funding towards malaria control, scaling up and directing interventions based on epidemiological evidence, and strengthen active malaria surveillance and response to reduce transmission and to begin considering elimination.

## 1. Introduction

Malaria continues to be a disease of major public health significance in Zambia despite recent successes in scaling up interventions and documented reductions in malaria burden among children [1–4]. The report article entitled “Achievements in Malaria Control: The Zambian Story 2000–2010” was published in 2010 by the Directorate of Public Health and Research of the Ministry of Health (MoH) in Zambia [2]. The publication indicates that in the 10–20 years leading up to the year 2000, relatively limited malaria prevention existed in the country and much of the activities were focused on treatment of malaria. This led to steady increase in the disease burden, with hospital admissions increasing from 8.8% in 1976 to over 20% in the 1990s. Accordingly, case fatality rates in hospitalized patients increased from 10.6 deaths per 1000 malaria admissions in 1976 to 51 deaths per 1000 malaria admissions in 1994 [5]. In 1999, approximately 3.46 million malaria cases were recorded for a population of 10.8 million inhabitants. The malaria case rate was 4- to 5-fold higher in children under 5 years of age compared to those above 5 years of age. The situation prompted the Zambian Government to place malaria as a priority area and clearly outlined it in both the National Health Strategic Plan and the National Development Plan [6–8]. In an effort to reduce the impact of malaria and contribute to the attainment of the Roll Back Malaria (RBM) targets and health related Millennium Development Goals (MDGs), malaria control measures using an integrated approach with evidence-based proven prevention, control and management interventions were reintroduced in Zambia [6–9].

Major malaria vectors in the country are *Anopheles gambiae* s.s. *An. arabiensis* and *An. funestus* s.s [10]. The predominant malaria parasite species is *Plasmodium falciparum*, with *Plasmodium malariae* and *Plasmodium ovale* accounting for less than 5 percent [11]. Zambia's initial National Malaria Control Strategic Plan covered the period from 2000 to 2005; the plan was updated for 2006 to 2010, setting ambitious goals to scale up a package of malaria interventions [7, 9]. The key malaria prevention, control and management strategies that Zambia took to mitigate the disease are: (1) vector control using indoor residual spraying (IRS) and promotion of ownership and use of insecticide-treated nets (ITNs); (2) malaria case management using effective diagnostics and lifesaving drugs-artemisinin-based combination therapy (ACTs); (3) control of malaria in pregnancy through intermittent presumptive treatment (IPTp) strategy; (4) information, education, and communication (IEC)/behavioural change communication (BCC) strategies.

The country has made great progress in the fight against malaria (Tables 1 and 2). The operational scale deployment of effective control tools has transformed the epidemiological profile from country-wide high endemicity to three distinct epidemiological strata: very low transmission and parasite prevalence of <1%, low transmission (1–10%), and persistent high transmission (>10%) [2–4]. Intermittent presumptive treatment in pregnancy (IPTp) uptake has reached the RBM target at 86% including uptake of two to three doses of IPTp representing 70% which is one of the highest in Africa [2–4] (Tables 1 and 3). The incidence of malaria has declined by 39% between 2006 and 2008, and a more than 60% decline in inpatient malaria cases between 2001 and 2008, in both under 5 and 5 to 15 year age groups [6–8, 12–14]. Parasite prevalence among children under five in Zambia declined from 22% to 16% in 2010 [2–4] (Table 1). 

The report aims at sharing with the rest of the malaria community the achievements made by the malaria control programme in Zambia and highlighting the need to maintain the thrust and the gains as the programme strives towards achieving universal coverage of evidence-based and proven interventions. Particularly, the need to scale up and direct interventions based on epidemiological and entomological evidence (including insecticide susceptibility and management of resistance) strengthens active malaria surveillance and response to reduce transmission, to address the epidemiological differences across the country, and utilize the evidence for ongoing refinement of policy and strategy and strengthens malaria control operations at provincial, district, and community levels in accordance with national policies based on decentralization programs to consolidate partnership and performance management in order to address human and financial resource needs, commodity requirements, and program action, as well as addressing the low utilisation and acceptance of interventions through increased advocacy, education, and communication for behaviour change.

The main findings or arguments of the report are that (1) sustaining high levels of transmission-reducing interventions is critical to the long-term success of malaria control and its future elimination; (2) a solid and predictable resource base is absolutely required for effective planning and efficient programme implementation; (3) mobilization and efficient coordination of partners have markedly contributed to the success of the malaria control efforts in Zambia; (4) advocacy, communication, and behavioural change are key for strengthened political will, national leadership, community ownership and involvement, and concerted efforts from all stakeholders; (5) all these aspects together could facilitate for the ultimate attainment of a malaria-free Zambia. 

Thus, the success that Zambia has achieved in malaria control can be attributed to the strong partnerships, increased resources, and evidence-based deployment of interventions in accordance with the national malaria control programme (NMCP) policy and strategic direction [15]. In light of enhanced advocacy and strengthened partnerships, there is unequivocally strong need for thorough evaluation of the performance of different aspects of the control programme. Herein we provide an in-depth evaluation of the strengths, weaknesses, and key issues of the report on the achievements of malaria control in Zambia. 

## 2. Review

**Figure 1:**
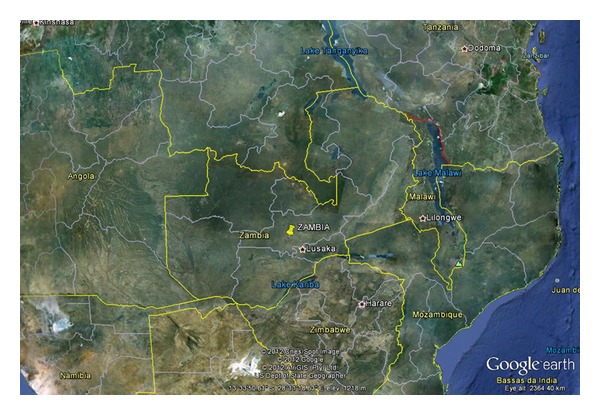
Map of Zambia showing the location of the neighbouring countries in Southern Africa.

The report is well written and attractively produced but there are some notable gaps. For example, the report does not give from the outset a clear background of the country's demographic and epidemiologic description. However, Zambia is situated in the Southern African region between 8° and 18° degrees south latitude and between 20° and 35° degrees east longitude with a population of approximately 13 million [16] in 10 provinces (Figure 1). There are three distinct seasons: a cool and dry season from April to August, a hot and dry season from August to November, and a warm and rainy season from November to April. Malaria is endemic with regular and moderate to high transmission across the entire country with a seasonal pattern of high transmission peaks between December and May coinciding with the rainy season [5]. 

There is a clear indication that the MoH implements a sector wide approach (SWAp) which harnesses the pooling of financial resources into the district basket funding leading to regular, predictable, and sustained flow of resources [17]. However, the report does not bring out strongly the challenges of the dwindling financial resources that have followed in the wake of diminishing donor support and the limited government funding for malaria control. Equally, most challenges are not addressed adequately but rather confined to specific interventions even when they relate to all aspects of malaria control. To illustrate, the lack of adequate competent human resource pool in the health sector necessary for driving forward the malaria control agenda is minimally addressed. The report only alludes to this challenge in relation to operational research and malaria case management and diagnosis. 

In the same vein, coordination and partnerships at district level that remains a major stumbling block to effective deployment of interventions received little attention, as indicated by the need to reinforce partnership engagement for IRS, particularly with the local authorities, at this level. Generally flaws in the supply chain management of commodities and equipment have resulted in delayed implementation of key preventive interventions and timely management of the disease. The report only mentions the need to collaborate with Medical Stores Limited and the reproductive health department to assure supplies of sulphadoxine-pyrimethamine (SP) for IPTp and streamline the distribution of SP to all ante natal clinic facilities. Most statements in the report are either not or are inadequately referenced. The success story could have been greatly enhanced if the foregoing shortfalls were addressed.

### 2.1. Policy and Strategic Direction

Since 2000, when the RBM initiative was launched in Zambia, the number of malaria programme partners has increased, translating into increased financial, technical, material, and human resources for malaria control. Zambia is fully committed to reducing the impact of malaria and contributing to the attainment of the Abuja Declaration, Millennium Development Goals (MDGs), and the RBM targets. The country has developed the National Health Strategic Plan 2011–2015, which is very critical to achieving the MDGs, and the sixth National Development Plan that focuses on malaria elimination as one of the key health priorities. A comprehensive National Malaria Control Strategic Plan from 2011 to 2015 including several intervention-specific guidelines has also been developed. 

### 2.2. Technical Interventions

Scaling up of malaria prevention and control programme interventions has been intensified by the MoH with substantial and important scores made towards achieving the health-related Millennium Development Goals (MDGs) and other key national achievements in relation to RBM targets. With assistance from valuable partners, strong leadership, and political will, the MoH has expanded the availability and access to ITNs with over seven million having been distributed since 2004, with increased ownership of ITNs from 38 percent (2006) to 64 percent in 2010 (Figures 3 and 4). Coverage of IRS has been scaled up from five initial districts in 2003 to 15 in 2006, 36 in 2008, 54 in 2010, and 72 in 2012 (Figure 5), and uptake of full dosing of IPTp has increased significantly from 59 percent (2006) to 70 percent 2010. Equally, maternal mortality rate per 100,000 population decreased markedly from 729 in 2001 to 591 in 2007. The government has ensured availability of malaria commodities such as diagnostic tools and efficacious drugs at all of public health facilities including at community level using community health workers for home management of malaria. 

Nevertheless, the publication covers the achievements of malaria control in Zambia broadly and yet with sufficient information to be useful to other control programmes that are intending to scale up interventions. It highlights the integrated approach that the national malaria control programme has implemented [6–9]. The strategy emphasizes a core set of evidence-based proven preventive and treatment interventions for malaria control [18, 19] including: ITNs with a vision of attaining universal coverage with all sleeping spaces in all households [13, 14, 20] (Figures 3 and 4); IRS to ensure that at least 80% of the targeted structures in IRS eligible districts are protected [21, 22]; case management and parasite detection to ensure that at least 80% of malaria patients to receive prompt and effective diagnosis and treatment within 24 hours of onset of symptoms [23, 24]; IPTp to ensure that at least 80% of pregnant women have access to the package of interventions (SP and ITN) to reduce the burden of malaria in pregnancy [6–8, 19]. 

### 2.3. Monitoring and Evaluation

The MoH has further conducted surveys and reviews to assess the impact of malaria prevention and control interventions. These include the Demographic and Health Survey [25, 26], Malaria Indicator Surveys [3, 8, 14], and the Malaria Programme Review [4]. The impact of Zambia's interventions is visible through the reduction of the annual number of malaria deaths by over 60 percent between 2000 and 2008 [1]; under five malaria deaths by 41 percent between 2006 and 2008; reduced severe anemia rates in children by 56 percent (2006–2010) (Table 1). According to the WHO assessment conducted in 2008, Zambia recorded a decline in malaria cases by 66 percent [1]. With this achievement, the country has surpassed targets set by (i) the Abuja Declaration by Heads of States in 2000 of reducing malaria illness and deaths by fifty percent by 2010, (ii) the RBM goal of reducing the global malaria burden by fifty percent by 2010.

One notable innovation worth grasping is the efficient utilization of supportive strategies to streamline uptake and purposeful deployment of key preventive and treatment tools. In Zambia, implementation of key malaria control interventions is augmented with cross-cutting supportive approaches. The report highlights an interactive advocacy, communication, and behaviour change to enhance utilization of interventions through promotion of appropriate care seeking behaviour [8]; viable operations research (OR) feeding into and providing timely and sound evidence to guide implementation of malaria control and inform policy decision making. Here a unique Zambia feature is coordination of the OR network, with strong collaborations of various local and international research institutions, whose information is shared with all stakeholders such as implementers, policy makers, funding agencies, and academic institutions. There is strong evidence-based monitoring and evaluation to facilitate for the documentation of progress made towards the achievement of goals and targets of the United Nations MDGs by 2015. Zambia also has solid, consistent, and coordinated policies and strategies for malaria control in place. This includes a comprehensive national malaria strategic plan for 2011–2015, policy guidelines for key interventions, and support services as well as budgeted annual work plans.

With the country-wide scaling up of vector control interventions, entomological monitoring and management of insecticide resistance is the major challenge [10]. In response to this, Zambia is again unique in developing a robust network of local Malaria Institute at Macha (MIAM), Zambia Integrated System Strengthening Programme (ZISSP), University of Zambia (UNZA), Tropical Disease Research Centre (TDRC), Zambia Environmental Management Agency (ZEMA), international-World Health Organization (WHO), Centres for Disease Control and Prevention (CDC), United States Agency for International Development (USAID), Malaria Transmission Consortium (MTC), Innovative Vector Control Consortium (IVCC), John Hopkins Malaria Research Institute (JHMRI), and the Liverpool School of Tropical Medicine (LSTM) entomology partners with clear terms of reference to consolidate and coordinate resistance monitoring and data collation to make recommendations for pesticide procurement [27].

### 2.4. Partnership and Coordination

The Ministry of Health leads malaria control efforts in Zambia through its National Malaria Control Centre (NMCC), provincial and district health offices and health facilities. Many multilateral agencies, nongovernmental organizations, research institutions, and community-based organizations are engaged in malaria control efforts throughout the country in implementing interventions, training health workers, and strengthening IEC/BCC. Increased community and private sector engagement coupled with strong partnership coordination is striking. Notably the national IRS program was built upon collaboration with Konkola Copper Mines (KCM), Mopani Copper Mines, and Zambia Sugar programs. The success of the malaria control can be ascribed to exceptional efforts towards establishment of strong partnership coordination; engaging community leaders and health workers as front line in the fight, an emphasis that echoes the mission of the ministry of health to provide quality health care as close to the family as possible; involvement of private sector to complement the public sector efforts and strengthening of malaria operations research to facilitate for evidence-based programming. The strengthening and coordination of partners under the stewardship and leadership of government has contributed to the increased and sustained number of multilateral, bilateral, national, faith-based, private sector, and community organizations. More specifically, the three-ones approach: one coordinating mechanism; one implementation plan, and one monitoring plan is largely responsible for the success in partner coordination, strengthening, and mobilization. 

Zambia has some unique stories to tell. One of the unique feature of the Zambian NMCP is the partnership with community-based organizations such asestablishment of the Zambia Malaria Foundation to operationalize the concept of an NGO umbrella group. This provided a forum to engage and coordinate with a very broad range of NGOs, from the Zambia Scouts Association (who used to help in the net retreatment campaigns) to the small youth and church groups and to business groups such as Rotary, as well as the Zambia Association of Chambers of Commerce and Industry (ZACCI). In addition, there has been an exceptional partnership with the HIV/AIDS programs. Both in information, education, and communication (IEC) and behaviour change communication (BCC) in support for ITNs targeting people living with HIV/AIDS (PLWHA) through home-based care groups such as the “Reaching HIV/AIDS Affected People with Integrated Development and Support” (RAPIDS) project. The Zambian programme was one of the first to really embrace integrated management of child illnesses (IMCI), then the first for “Fresh Air,” NGO coordination, the first for nation-wide roll out of ACT (Coartem), the second (after the small district distribution in Ghana by IFRC) for a mass-free distribution of LLINs, and the first for the integrated vector management strategy (IVM) policy [18].

The NMCC is pivotal in providing technical guidance, leadership, and coordination of malaria control and preventive activities. It ensures full participation and involvement of partners in the development of key documents: strategic plans, annual action plans, and policy guidelines through intervention specific multisectoral technical working groups for vector control; information, education, and communication; monitoring, evaluation, and operations research. 

### 2.5. Financing and Human Resources

**Figure 2:**
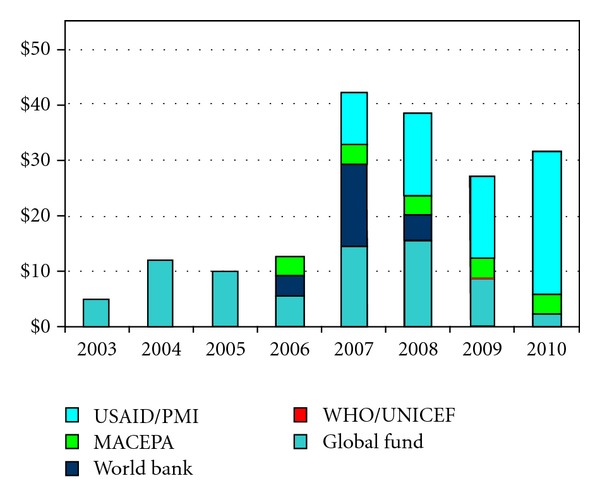
Zambia: external funding (in millions, US$) for the Zambia Malaria Control Programme, 2003–2010. Source: Roll Back Malaria Progress and Impact Series—focus on Zambia.

**Figure 3:**
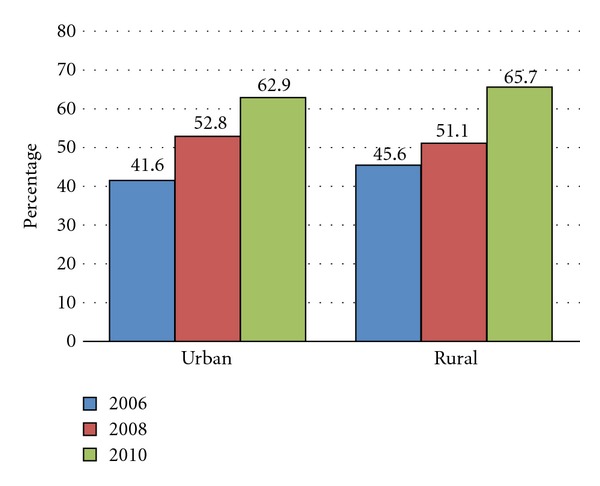
ITN used by children under age of five years in rural and urban areas (source: *Zambia Malaria Indicator Surveys 2006–2010*) [3, 8].

**Figure 4:**
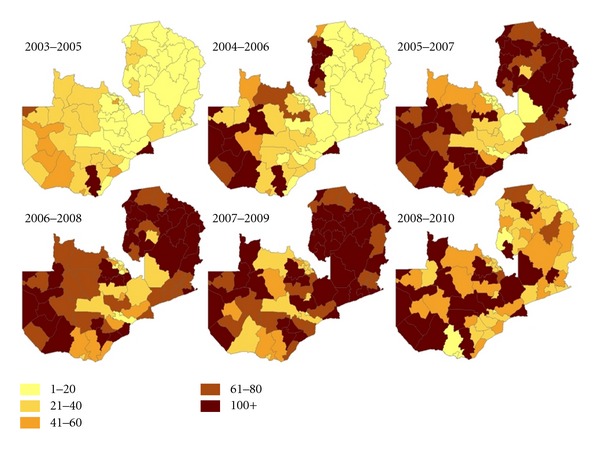
District level ITN coverage expressed as percentage of 3 ITNs distributed per district household estimate, by three-year intervals. (Source: Zambia Malaria Indicator Surveys 2006–2010) [3, 8].

**Figure 5:**
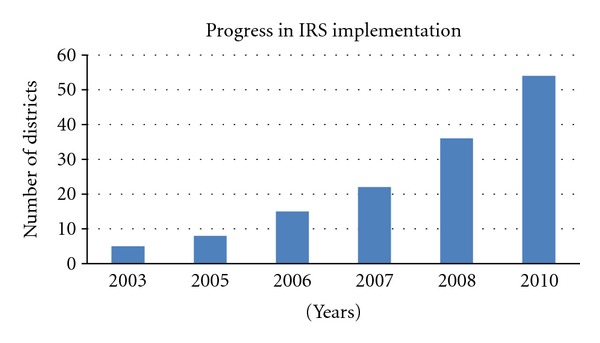
Progressive scale-up of indoor residual spraying from 2003 to 2010.

The NMCP receives financial and technical support from a variety of organizations to enable a coordinated approach to scaling-up interventions and tracking progress. Partners providing the largest financial contributions to malaria control efforts in Zambia apart from government includes the World Bank, Global Fund to fight HIV, TB, and malaria (GFATM), and the United States' President's Malaria Initiative (PMI) (Figure 2). The challenge of limited human resources for malaria control is circumvented by increased capacity building at provincial and district levels and by increasing collaboration with other implementing partners.

As funding for malaria control gets tighter it is important for countries to demonstrate “the business case” that investments in malaria control reap economic and social benefits. Zambia has solid evidence from the private sector that is, the programme has a unique collaboration with the private sector; such as the mining industry; Konkola Copper Mines, Mopani Copper Mines, and the agricultural sector; Zambia Sugar company programs that have shown a “positive return for investment” for their workplace malaria programs. There has also been a lot of engagement with Zambia Association of Chambers of Commerce and Industry (ZACCI) to try to expand “the business case” to other sectors.

## 3. Conclusions

The malaria control programme in Zambia has made great achievements in its control efforts through provision of high coverage of malaria prevention and curative services. The success can be attributed to the strong partnerships including community engagement, increased resources, and evidence-based deployment of key technical and supportive interventions in accordance with the national malaria control programme policy and strategic direction. The country offers some unique models and experiences that could really benefit other programmes in the region. Community level integrated entomological and case surveillance, prompt effective treatment, and sustained high levels of contemporary malaria prevention tools, are pivotal to the long-term success of malaria control and future malaria elimination. However, there is great need for increased resource mobilization by broadening the partnership base and increasing the government commitment to malaria control. 

## Figures and Tables

**Table 1:** Benchmarking change in Zambia.

Indicator	DHS 2001/2002	MIS 2006	DHS 2007	MIS 2008	MIS 2010
Percentage of households with at least one insecticide-treated net (ITN)	14	38	53	62	64
Percentage of households with at least one ITN per sleeping space	N/A	N/A	N/A	33	34
Percentage of households receiving IRS in the previous 12 months among all households	N/A	10	N/A	15	23
Percentage of households covered by at least one ITN or recent IRS	N/A	43	N/A	68	73
Percentage of children ages 0–59 months who slept under an ITN the previous night	7	24	29	41	50
Percentage of pregnant women (PW) who slept under an ITN the previous night	8	25	33	43	46
Percentage of household members who slept under an ITN the previous night	N/A	19	N/A	34	42
Percentage of PW who took any preventive antimalarial drug during pregnancy	36	85	87	88	89
Percentage of PW who received 2 doses of intermittent preventive treatment during pregnancy	N/A	59	66	66	70
Percentage of children ages 0–59 months with severe anaemia (Hb < 8 g/dL)	N/A	14	N/A	4	9
Percentage of children ages 0–59 months with malaria parasitaemia	N/A	22	N/A	10	16
Percentage of women ages 15–49 years who recognize fever as a symptom of malaria	N/A	65	N/A	71	75
Percentage of women ages 15–49 years who reported mosquito bites as a cause of malaria	N/A	80	N/A	85	85
Percentage of women ages 15–49 years who reported mosquito nets as a prevention method	N/A	78	N/A	81	82

Source of data: DHS, MIS, and reports (2001 to 2010).

**Table 2:** Changes in child mortality rates in 2001/02 and 2007.

Indicator	2001/02 DHS	2007 DHS	Percent Change
Infant mortality	95	70	−26%
Neonatal mortality	37	34	−8%
Post natal mortality	58	36	−38%
Child mortality (1–4 yrs)	81	52	−36%
Under 5 mortality	168	119	−29%

Source: Zambia Demographic and Health Survey, 2001/2 and 2007.

**Table 3:** Summary of progress in MIP interventions.

Indicator	DHS 2001/2002	MIS 2006	MIS 2008	MIS 2010
Percentage of pregnant women (PW) who slept under an ITN the previous night	7.9	24.5	43.2	45.9
Percentage of PW who took any preventive antimalarial drug during pregnancy	35.8	85.3	88.1	89.0
Percentage of PW who received 2 doses of intermittent preventive treatment during pregnancy	N/A	58.9	66.1	70.2

Source: Zambia Demographic and Health Survey, 2001/2, MIS; 2006, 2008, and 2010.
